# Regional disparities and hospital performance patterns in acute stroke reperfusion: a multicenter study in Sichuan Province, China

**DOI:** 10.3389/fneur.2026.1837068

**Published:** 2026-07-01

**Authors:** Shu Liu, Rong Li, Yan Liu, Haitao Zhang, Rong Hu, Guangyu Zhong, Hua Liu

**Affiliations:** Department of Neurology, The Affiliated Hospital of Southwest Jiaotong University, The Third People's Hospital of Chengdu, Chengdu, China

**Keywords:** acute ischemic stroke, endovascular treatment, intravenous thrombolysis, regional disparities, reperfusion therapy

## Abstract

**Introduction:**

Timely reperfusion is critical to improving outcomes in acute ischemic stroke (AIS). However, disparities in treatment quality and accessibility persist across hospital levels, regions, and organizational types in China. This study assessed real-world performance of reperfusion care in Sichuan Province.

**Methods:**

We conducted a retrospective multicenter study across 33 hospitals in Chengdu from 2021 to 2024. Key process and outcome indicators—including door-to-needle time (DNT), door-to-puncture time (DPT), puncture-to-recanalization time (PRT), intravenous thrombolysis (IVT) within 4.5 h, endovascular treatment (EVT) within 6 h, and in-hospital mortality after EVT—were compared across hospital grades, geographic locations, and stroke center status. K-means clustering was used to explore hospital-level performance patterns based on reperfusion indicators.

**Results:**

Door-to-needle time and IVT rates remained relatively high and stable. In contrast, PRT compliance and EVT accessibility showed notable variation. Tertiary A-level hospitals, urban hospitals, and certified stroke centers were associated with higher observed performance in several indicators. EVT-related mortality showed a transient observed spike in non-tertiary A-level and non-stroke center hospitals in 2023. Exploratory cluster analysis identified three hospital-level performance patterns, suggesting persistent disparities in capacity, access, and EVT-related in-hospital outcomes.

**Conclusion:**

Our findings highlight institutional and regional inequities in AIS reperfusion care. Stroke center status was associated with higher observed efficiency, accessibility, and EVT-related in-hospital outcome indicators, although residual confounding cannot be excluded. To promote equitable, high-quality stroke care in western China, strategic investments are needed in infrastructure, workforce training, and networked stroke systems, particularly for non-tertiary A-level and non-stroke center hospitals.

## Introduction

Stroke remains a leading cause of morbidity, mortality, and long-term disability worldwide. According to the Global Burden of Disease Study 2019, the incidence and prevalence of stroke in low- and middle-income countries, including China, have been increasing over the past decades (1). Stroke is also the leading cause of death among urban and rural residents in China, and ischemic stroke (IS) accounts for about 80% of all strokes, posing a substantial health and economic burden on individuals, families, and healthcare systems (2). With an aging population and increasing prevalence of vascular risk factors such as hypertension, diabetes, and dyslipidemia, the burden of IS is projected to further escalate in the coming decades.

Timely reperfusion therapy remains the cornerstone of acute ischemic stroke (AIS) management. For patients with AIS, intravenous thrombolysis (IVT) with plasminogen activator (rt-PA) is effective within 4.5 h of onset, and endovascular thrombectomy (EVT) within 6 h is particularly beneficial for those with anterior circulation large vessel occlusion (3). Both therapies have been shown to improve functional recovery significantly and rely heavily on the efficiency of in-hospital stroke workflows—such as door-to-needle time (DNT), door-to-puncture time (DPT), and puncture-to-recanalization time (PRT)—which directly impact clinical outcomes (4, 5).

To improve stroke treatment capacity and reduce treatment delays, China has implemented several national-level initiatives, including the establishment of a hierarchical stroke center network, the construction of the national Stroke Center Certification System, and the launch of the China Stroke Center Alliance (CSCA) (6). Furthermore, the development of regional “Stroke Emergency Map” and prehospital triage protocols has promoted a coordinated care model integrating tertiary A-level and non-tertiary A-level hospitals, aiming to optimize stroke care accessibility and equality across different regions and hospital levels (7).

Chengdu, the capital city of Sichuan Province in western China, is a major national economic center with a population exceeding 21 million and administrative jurisdiction over 20 districts, county-level cities, and counties. Despite significant advancements in stroke care infrastructure in recent years, substantial disparities in reperfusion treatment capacity may persist across hospitals of different levels and geographic regions within the city. For example, a 2023 national analysis involving 109 tertiary hospitals in China reported wide variations in adherence to guideline-recommended stroke quality indicators—such as DNT and timely EVT—directly influencing functional outcomes (8). Similarly, a 2025 nationwide hospital-based study revealed significant rural–urban differences in AIS management, including lower rates of IVT and longer treatment delays in rural hospitals (9). While these national-level studies highlight substantial inter-hospital and regional disparities in stroke care across China, they are primarily based on aggregated data and do not capture heterogeneity within a single metropolitan healthcare system. In particular, variations across hospital levels, geographic locations, and stroke center certification status within an individual city remain insufficiently characterized. To fill this gap, we conducted a comprehensive assessment using data from 33 hospitals within Chengdu. By systematically analyzing key reperfusion process indicators—including DNT, DPT, PRT, and treatment accessibility metrics (e.g., IVT within 4.5 h and EVT within 6 h)—this study aims to provide new insights into real-world stroke care performance across a major urban region. The findings are intended to identify current strengths, highlight persistent gaps, and inform targeted quality improvement efforts within regional stroke systems.

## Methods

### Participants

This was a multicenter, retrospective, observational study conducted in Chengdu, Sichuan Province, China. Data were collected from 33 hospitals of varying levels and regions between January 2021 and December 2024. Patients diagnosed with AIS were included based on clinical symptoms and neuroimaging findings, following the diagnostic criteria established by the American Heart Association/American Stroke Association (AHA/ASA) (10). The participating hospitals comprised both tertiary A-level hospitals (i.e., the highest level in China’s three-tier hospital classification system, characterized by comprehensive clinical services, advanced medical technology, and strong capacity for stroke care) and non-tertiary A-level hospitals (i.e., tertiary B-level or secondary-level institutions with more limited resources and interventional capability). Additionally, hospitals were also stratified by stroke center certification status. Certified stroke centers—including both advanced and primary stroke centers—were defined according to national standards established by the National Health Commission and the China Stroke Prevention Project Committee (CSPPC). These standards include requirements for dedicated stroke teams, round-the-clock neuroimaging, thrombolysis and EVT capability, and minimum treatment volume thresholds (11). For the purposes of this study, advanced and primary stroke centers were analyzed as a single group (stroke centers), and hospitals without certification were designated as non-stroke centers. Among the 33 hospitals, 26 were certified stroke centers and 7 were non-stroke centers. In total, 24 were tertiary A-level hospitals and 9 were non-tertiary A-level hospitals, distributed across urban and suburban areas of Chengdu. This hospital sample reflects a diverse cross-section of the region’s acute stroke care network in terms of capacity, infrastructure, and geographic accessibility. All data were collected retrospectively through the provincial stroke quality control platform. As the study used de-identified retrospective data, informed consent was waived. The study protocol was approved by the Ethics Committee of the Third People’s Hospital of Chengdu.

### Data collection

Data collection focused on key performance indicators reflecting the efficiency, accessibility, and EVT-related in-hospital outcomes of reperfusion therapy for AIS. Specifically, process efficiency indicators included DNT, DPT, and PRT. Guidelines recommend a DNT of ≤60 min for patients receiving IVT, a DPT within 90 min for those undergoing EVT, and a PRT with an ideal target of ≤30 min and an upper limit of 60 min from groin puncture to successful reperfusion (12–14). Accessibility indicators included the proportion of patients receiving IVT within 4.5 h of symptom onset and the proportion receiving EVT within 6 h. The IVT within 4.5 h rate was calculated as the number of AIS patients receiving IVT within 4.5 h divided by the total number of AIS patients recorded in the corresponding subgroup-year cell. Similarly, the EVT within 6 h rate was calculated as the number of AIS patients undergoing EVT within 6 h divided by the total number of AIS patients recorded in the corresponding subgroup-year cell. In contrast, in-hospital mortality following EVT was calculated only among EVT-treated patients, using the number of in-hospital deaths after EVT as the numerator and the total number of EVT-treated patients as the denominator. In-hospital mortality following EVT was analyzed as an in-hospital outcome measure among EVT-treated patients. Importantly, this outcome was available only among patients who underwent EVT and therefore should not be interpreted as an overall outcome measure for the entire AIS cohort or for all reperfusion-treated patients. All variables were extracted from the provincial stroke quality control database using standardized definitions and calculation formulas based on national stroke care guidelines. The provincial stroke quality control database did not capture post-discharge outcomes, including discharge disposition, 90-day modified Rankin Scale (mRS), stroke recurrence, readmission, or long-term mortality. Other clinically important safety indicators, such as symptomatic intracranial hemorrhage (sICH), IVT-related complications, and procedure-related complications, were also unavailable in the current dataset. Data entry was performed by trained personnel, and data quality was centrally monitored to ensure completeness and accuracy.

### Statistical analyses

All statistical analyses were conducted using SPSS version 26.0 (IBM Corp., Armonk, NY, United States) and R version 4.5.1 (R Foundation for Statistical Computing, Vienna, Austria). Continuous variables were presented as means and standard deviations (*x̄* ± *s*), while categorical variables were expressed as frequencies and percentages. Group comparisons between hospital levels (tertiary A-level vs. non-tertiary A-level), geographic regions (urban vs. suburban), and stroke center designation (stroke center vs. non-stroke center) were performed using the chi-square test or Fisher’s exact test, as appropriate. Temporal trends over the four-year period (2021–2024) were assessed using the Cochran-Armitage trend test for binary outcomes. To explore hospital-level patterns in stroke care performance, K-means clustering was performed based on six standardized indicators: DNT compliance rate, DPT compliance rate, PRT compliance rate, intravenous thrombolysis rate within 4.5 h (IVT_4.5 h), endovascular therapy rate within 6 h (EVT_6h), and in-hospital mortality after EVT. All indicators were standardized before clustering to ensure comparability across variables with different scales. To improve reproducibility and reduce the influence of random initialization, K-means clustering was performed using a fixed random seed and multiple random starts. The number of clusters was evaluated using the elbow method based on total within-cluster sum of squares and silhouette analysis based on average silhouette width. Clustering solutions with different numbers of clusters were explored, and a three-cluster solution was selected because it provided a reasonable balance between statistical separation and clinical interpretability. Cluster-level means were used to generate radar plots for visual comparison across hospital performance patterns. Given the limited number of hospitals and the exploratory nature of this analysis, the cluster results should be interpreted as hypothesis-generating rather than as a definitive structural classification of hospitals. All statistical tests were two-tailed, and a *p*-value < 0.05 was considered statistically significant. All group comparisons were conducted at the hospital-indicator level and were not adjusted for patient-level case-mix variables. Because individual-level variables such as age, NIHSS score, comorbidities, LVO status, onset-to-door time, transfer status, and imaging characteristics were unavailable, the analyses should be interpreted as descriptive and exploratory rather than causal or risk-adjusted comparative assessments.

## Results

### Performance of reperfusion treatment capacity indicators

The performance of key reperfusion treatment capacity indicators was analyzed across different years, hospital levels, geographic regions, and stroke center designation. Specifically, compliance rates were defined as the proportion of patients meeting guideline-recommended thresholds: DNT ≤ 60 min for IVT, DPT ≤ 90 min for EVT, and PRT ≤ 60 min for EVT.

**Figure 1:**
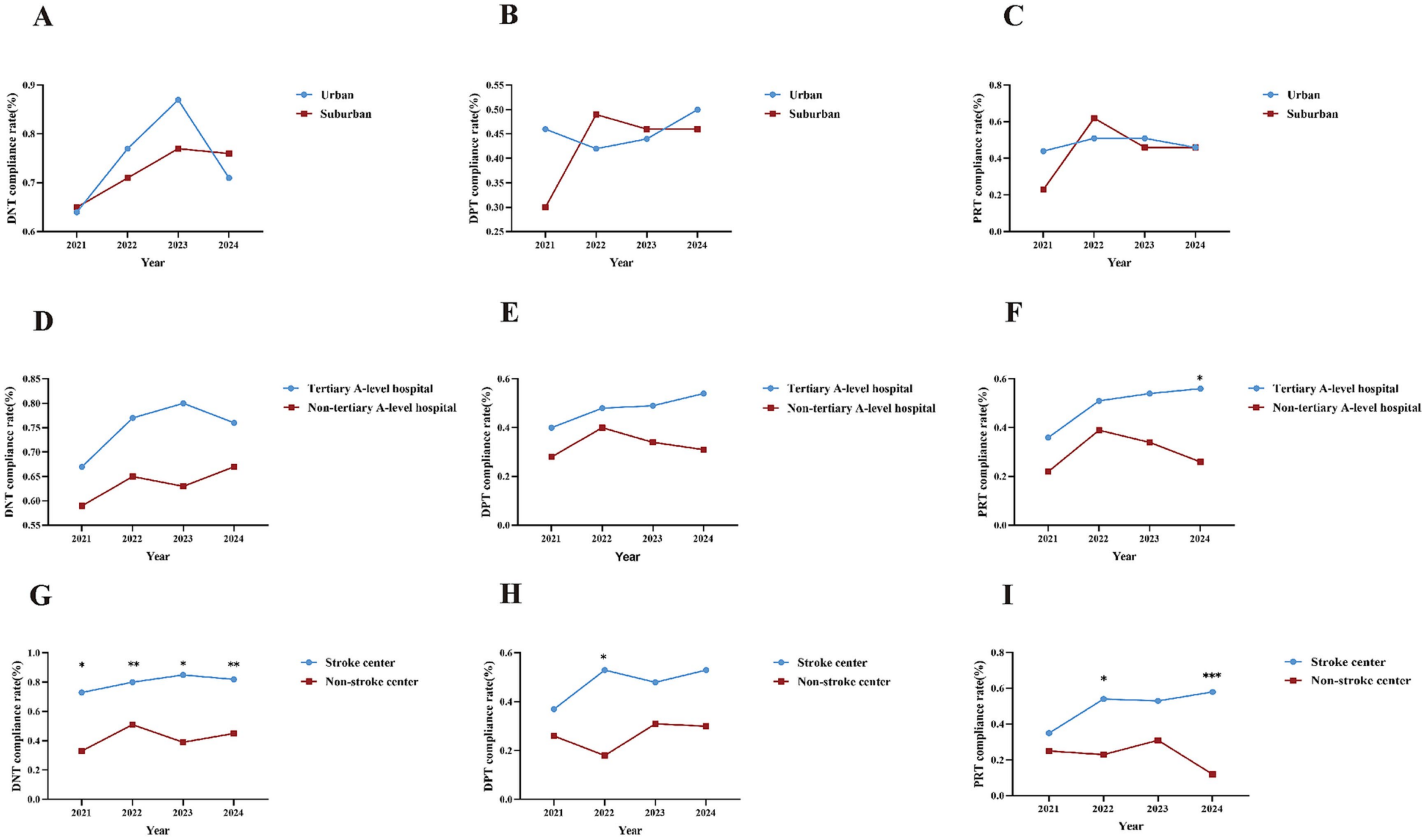
Temporal trends in compliance rates of reperfusion treatment indicators stratified by different hospital categories (2021–2024). **(A–C)** Compliance rates for DNT, DPT, and PRT by geographic region (urban vs. suburban). **(D–F)** Corresponding compliance rates stratified by hospital level (tertiary A-level vs. non-tertiary). **(G–I)** Corresponding compliance rates stratified by stroke center designation (stroke center vs. non-stroke center). Asterisks indicate statistically significant differences (*p < 0.05, **p < 0.01, ***p < 0.001). DNT, door-to-needle time; DPT, door-to-puncture time; PRT, puncture-to-recanalization time.

From 2021 to 2024, the overall DNT compliance rate remained relatively high and stable, ranging from 0.65 in 2021 to a peak of 0.74 in both 2022 and 2024. No statistically significant differences were observed between urban and suburban hospitals, nor between tertiary A-level and non-tertiary A-level hospitals over the four-year period (Figures 1A,D). However, when stratified by stroke center designation, stroke centers showed higher observed DNT compliance than non-stroke centers, with the difference reaching statistical significance each year (Figure 1G). For DPT compliance, a gradual improvement was observed over time, increasing from 0.37 in 2021 to 0.48 in 2024. Differences between geographic regions and hospital levels were minor and not statistically significant (Figures 1B,E), although stroke centers demonstrated significantly higher DPT compliance in 2022, suggesting a potential advantage in organizational readiness or team coordination (Figure 1H). PRT compliance also showed progressive improvement, from 0.33 in 2021 to 0.49 in 2024. More notable disparities emerged in this metric: tertiary A-level hospitals showed consistently higher PRT compliance in 2023 and 2024 compared to non-tertiary A-level institutions, indicating potential differences in technical capability or procedural efficiency. Likewise, stroke centers showed significantly higher observed PRT compliance across multiple years, especially in 2024, which may reflect differences associated with stroke center certification, although residual confounding by hospital resources, workflow organization, and case mix cannot be excluded (Figures 1C,F,I).

These findings, summarized in Table 1 and Figure 1, suggest that disparities in time-sensitive process metrics are more pronounced between stroke centers and non-stroke centers, while differences across geographic regions and hospital administrative levels were less consistent and often not statistically significant. The pronounced gaps between stroke centers and non-stroke centers across all three indicators further highlight the critical role of certified infrastructure and multidisciplinary coordination in delivering high-quality acute stroke care.

**Table 1:** Compliance rates of reperfusion treatment indicators from 2021 to 2024, stratified by different hospital categories.

Year	Overall	Urban (%)	Suburban (%)	*P*-value	Tertiary A-level hospital	Non-tertiary A-level hospital	*P*-value	Stroke center (%)	Non-stroke center (%)	*P*-value
DNT										
2021	0.65	0.64	0.65	0.91	0.67	0.59	0.49	0.73	0.33	0.02
2022	0.74	0.77	0.71	0.5	0.77	0.65	0.21	0.8	0.51	0.006
2023	0.75	0.87	0.77	0.46	0.80	0.63	0.17	0.85	0.39	0.01
2024	0.74	0.71	0.76	0.54	0.76	0.67	0.27	0.82	0.45	0.006
DPT										
2021	0.37	0.46	0.3	0.25	0.4	0.28	0.48	0.37	0.26	0.975
2022	0.46	0.42	0.49	0.53	0.48	0.4	0.56	0.53	0.18	0.02
2023	0.44	0.44	0.46	0.96	0.49	0.34	0.26	0.48	0.31	0.26
2024	0.48	0.50	0.46	0.7	0.54	0.31	0.06	0.53	0.3	0.09
PRT										
2021	0.33	0.44	0.23	0.08	0.36	0.22	0.32	0.35	0.25	0.5
2022	0.48	0.51	0.62	0.64	0.51	0.39	0.4	0.54	0.23	0.03
2023	0.48	0.51	0.46	0.67	0.54	0.34	0.18	0.53	0.31	0.29
2024	0.49	0.46	0.46	0.93	0.56	0.26	0.02	0.58	0.12	<0.001

This table summarizes the yearly overall compliance rates for three key indicators: DNT (≤60 min), DPT (≤90 min), and PRT (≤60 min), along with subgroup comparisons by hospital level (tertiary A-level vs. non-tertiary A-level), geographic region (urban vs. suburban), and stroke center designation (stroke center vs. non-stroke center). DNT, door-to-needle time; DPT, door-to-puncture time; PRT, puncture-to-recanalization time.

### The accessibility of reperfusion therapy

We analyzed the accessibility of reperfusion therapy across 33 hospitals in Chengdu from 2021 to 2024, focusing on two key indicators: the proportion of AIS patients who received IVT within 4.5 h of symptom onset, and those who underwent EVT within 6 h.

**Figure 2:**
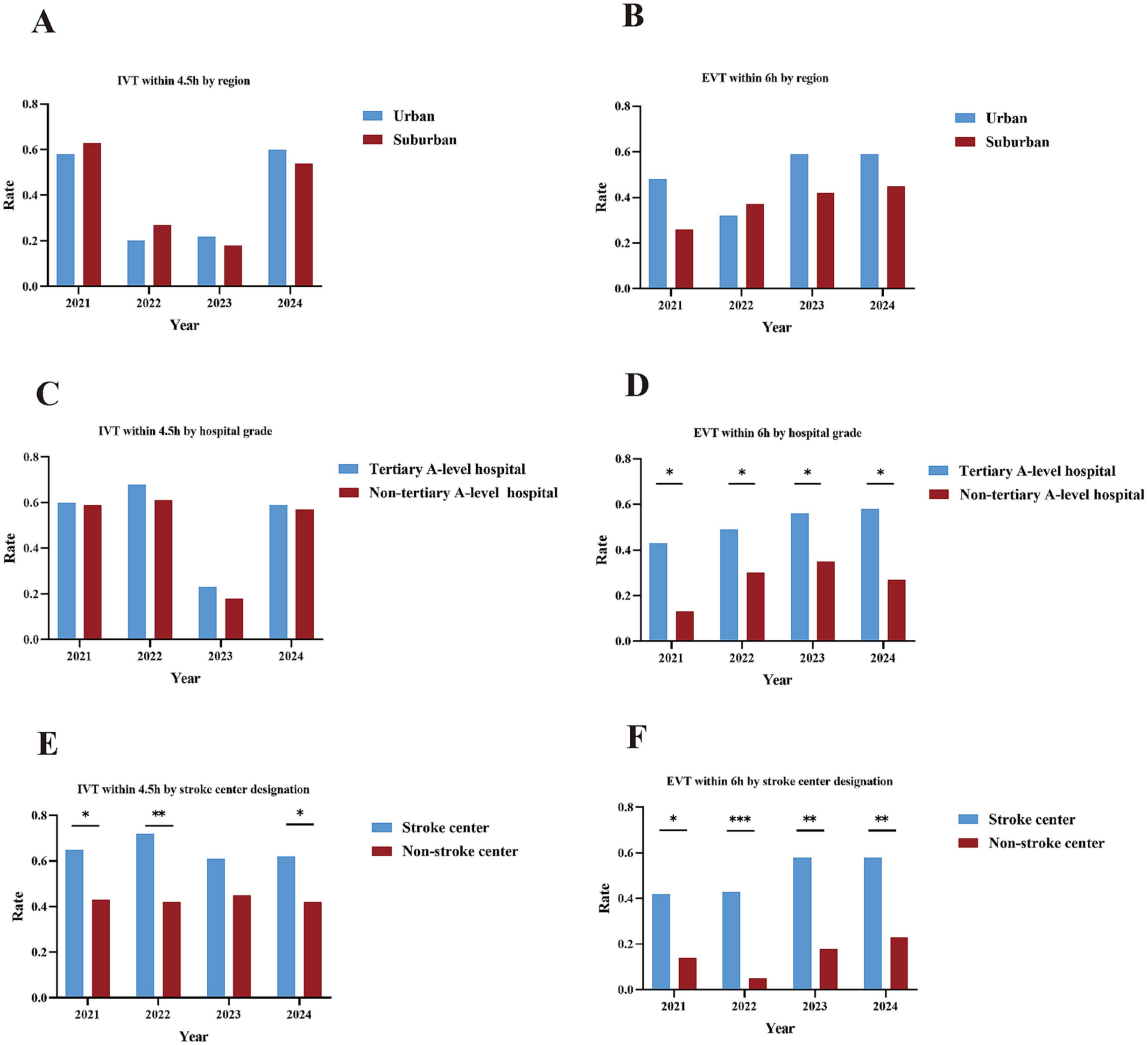
Accessibility of reperfusion therapy by region, hospital level and stroke center designation (2021–2024). **(A,C,E)** Annual rates of IVT within 4.5 h by region, hospital level and stroke center designation. **(B,D,F)** Annual rates of EVT within 6 h by region, hospital level and stroke center designation. Asterisks indicate statistically significant differences (*p < 0.05, **p < 0.01, ***p < 0.001). IVT: intravenous thrombolysis; EVT: endovascular thrombectomy.

The overall IVT rate remained relatively stable, ranging between 0.57 (2024) and 0.66 (2022). No statistically significant differences were observed between urban and suburban hospitals in any given year (Figure 2A). Similarly, tertiary A-level and non-tertiary A-level hospitals demonstrated comparable IVT rates, with no consistent trends of disparity (Figure 2C). Interestingly, IVT rates were slightly lower in urban hospitals in 2021 and 2022, potentially reflecting interannual variation in prehospital delays or patient selection. In contrast, EVT accessibility showed more pronounced variation (Figures 2B,D,F). From 2021 to 2024, the overall EVT rate increased from 0.36 to 0.51. Tertiary A-level hospitals consistently reported significantly higher EVT rates than non-tertiary A-level hospitals throughout all 4 years. A similar pattern was observed in comparisons between urban and suburban hospitals, although the differences were less consistent and not always statistically significant. To further elucidate institutional disparities, we additionally categorized hospitals into stroke centers and non-stroke centers based on national certification criteria. Stroke centers showed higher observed accessibility to both IVT and EVT compared with non-stroke centers across all years. This disparity persisted over time, with EVT rates in non-stroke centers remaining below 0.25 through 2024, which may reflect differences in interventional capability, referral pathways, patient triage, or case mix.

These findings underscore the critical role of designated stroke centers in facilitating timely reperfusion, particularly in the context of EVT, and highlight an urgent need for capacity-building efforts in non-stroke center hospitals to reduce accessibility gaps across the region (Table 2 and Figure 2).

**Table 2:** Rates of IVT within 4.5 h and EVT within 6 h, stratified by region, hospital level and stroke center designation (2021–2024).

Year	Treatment type	Overall (%)	Urban (%)	Suburban (%)	*P*-value	Tertiary A-level hospital	Non-tertiary A-level hospital	*P*-value	Stroke center (%)	Non-stroke center (%)	*P*-value
2021	IVT within 4.5 h	0.6	0.58	0.63	0.62	0.6	0.59	0.58	0.65	0.43	0.045
	EVT within 6 h	0.36	0.48	0.26	0.07	0.43	0.13	0.04	0.42	0.14	0.02
2022	IVT within 4.5 h	0.66	0.2	0.27	0.76	0.68	0.61	0.56	0.72	0.42	0.003
	EVT within 6 h	0.35	0.32	0.37	0.57	0.49	0.3	0.04	0.43	0.05	<0.001
2023	IVT within 4.5 h	0.58	0.22	0.18	0.21	0.23	0.18	0.32	0.61	0.45	0.07
	EVT within 6 h	0.5	0.59	0.42	0.13	0.56	0.35	0.04	0.58	0.18	0.001
2024	IVT within 4.5 h	0.57	0.6	0.54	0.36	0.59	0.57	0.77	0.62	0.42	0.016
	EVT within 6 h	0.51	0.59	0.45	0.19	0.58	0.27	0.03	0.58	0.23	0.004

This table summarizes the annual rates of AIS patients receiving IVT within 4.5 h and EVT within 6 h of onset. Comparisons are presented for urban vs. suburban hospitals, tertiary A-level vs. non-tertiary A-level hospitals and stroke center vs. non-stroke center. *p*-values indicate the significance of subgroup differences for each year. IVT, intravenous thrombolysis; EVT, endovascular thrombectomy.

### In-hospital mortality following EVT

We evaluated in-hospital mortality following EVT as an in-hospital outcome measure among EVT-treated patients. Mortality was defined as the proportion of patients who died during hospitalization among those who underwent EVT. Because this indicator was calculated only among EVT-treated patients, the mortality analysis should not be interpreted as the mortality risk of all AIS patients treated at each hospital, nor as a comprehensive measure of reperfusion effectiveness. Rather, it provides a hospital-level descriptive measure of severe in-hospital outcomes among patients undergoing endovascular therapy. Analyses were stratified by year, hospital level (tertiary A-level vs. non-tertiary A-level), geographic region (urban vs. suburban), and stroke center status (stroke center vs. non-stroke center).

From 2021 to 2024, in-hospital mortality rates remained relatively low across most subgroups. Tertiary A-level hospitals demonstrated consistent mortality rates, ranging from 0.05 in 2024 to a peak of 0.11 in 2022, with a gradual decline in recent years. In contrast, non-tertiary A-level hospitals showed substantial variation, notably reaching a high of 0.27 in 2023, before decreasing to 0.04 in 2024. A similar trend was observed across geographic regions: urban hospitals maintained relatively stable and low mortality, whereas suburban hospitals showed a sharp increase in 2023, followed by improvement in 2024. When comparing stroke centers versus non-stroke centers, an interesting pattern emerged. Stroke centers exhibited mortality rates ranging from 0.06 to 0.11, which, although modest, were generally higher than those observed in non-stroke centers during the first 2 years. This may reflect the more complex or severe cases managed at certified stroke centers. However, in 2023, non-stroke centers showed a marked increase in EVT-related in-hospital mortality. Given the lack of patient-level adjustment and the potential influence of denominator size, this finding should be interpreted as an unadjusted descriptive mortality signal rather than evidence of worse hospital performance. Potential explanations include differences in case mix, referral of higher-risk patients, variation in baseline stroke severity, or random fluctuation due to a small number of EVT cases. By 2024, mortality in non-stroke centers decreased to 0.01; however, the reason for this decline cannot be determined from the current hospital-level dataset.

**Figure 3:**
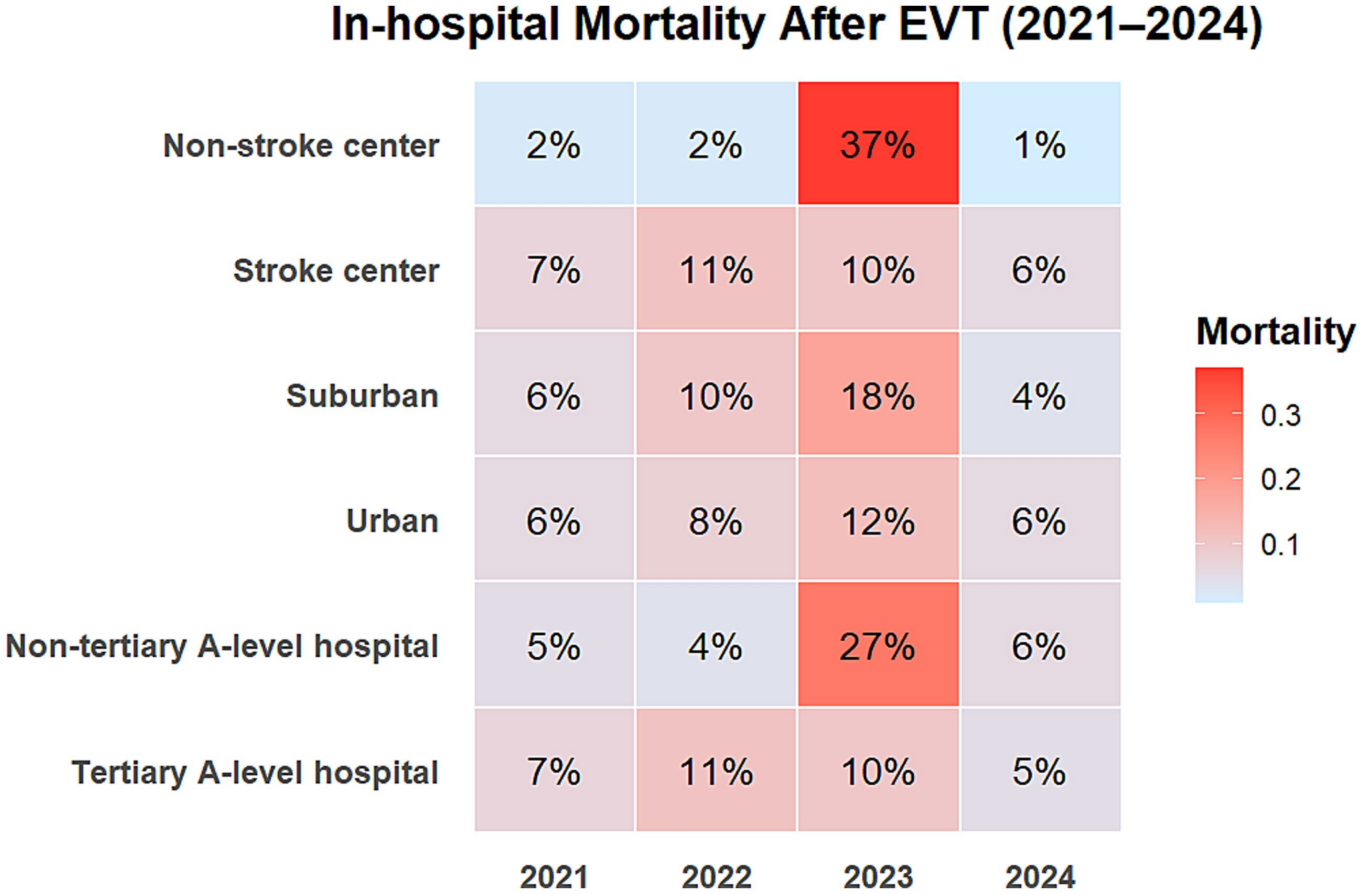
In-hospital mortality following EVT, stratified by hospital level, region and stroke center designation (2021–2024). This heatmap visualizes annual in-hospital mortality rates among AIS patients receiving EVT, categorized by geographic region (urban vs. suburban), hospital level (tertiary A-level vs. non-tertiary A-level) and stroke center designation (stroke center vs. non-stroke center). Mortality is expressed as the proportion of EVT patients who died during hospitalization in each subgroup. Color intensity corresponds to mortality rates, with darker red indicating higher rates. AIS, acute ischemic stroke; IVT, intravenous thrombolysis; EVT, endovascular thrombectomy.

Taken together, these findings suggest that stroke centers showed relatively more stable EVT-related in-hospital mortality over time. However, given the lack of patient-level risk adjustment, this pattern should not be attributed solely to hospital expertise, workflow organization, or care processes. The wide fluctuations in mortality observed in non-tertiary and non-stroke centers may indicate potential targets for quality improvement, including enhanced training, technical support, and procedural standardization, but may also reflect case-mix differences, referral patterns, procedural complexity, or small denominators. These findings need to be confirmed in future risk-adjusted analyses (Figure 3).

### Exploratory hospital-level performance patterns based on reperfusion process and outcome indicators

**Figure 4:**
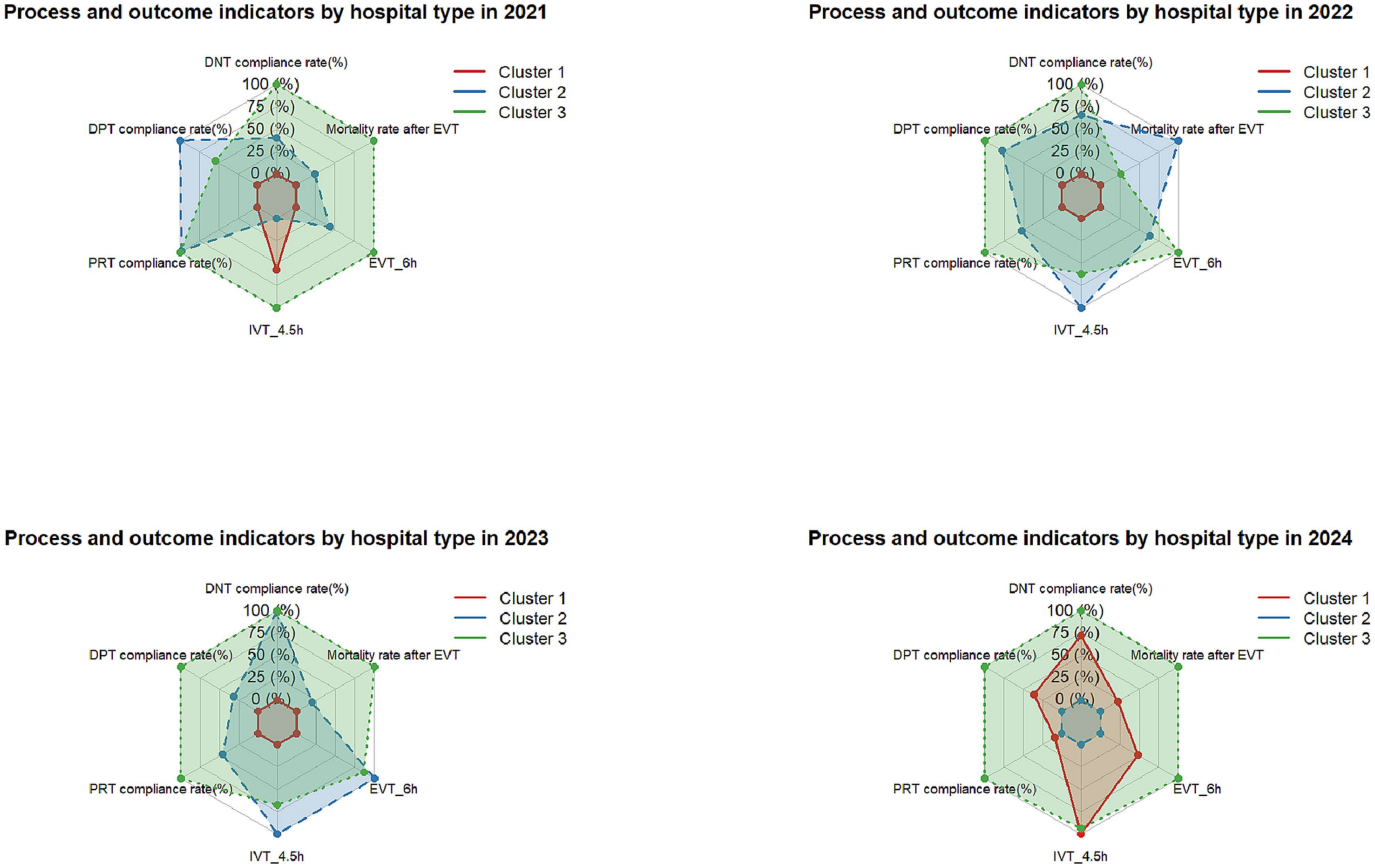
Cluster classification of hospitals based on reperfusion process and outcome indicators from 2021 to 2024. Radar plots display the performance of 33 hospitals clustered into three groups annually using K-means analysis of six indicators: compliance rates for DNT, DPT, and PRT; rates of intravenous thrombolysis within 4.5 h and endovascular therapy within 6 h; and in-hospital mortality after endovascular therapy. Cluster 3 (green): hospitals with higher observed process adherence, greater accessibility, and lower EVT-related in-hospital mortality, mostly tertiary centers. Cluster 2 (blue): hospitals with intermediate observed performance across indicators. Cluster 1 (red): hospitals with lower observed performance across several indicators, mainly non-tertiary centers. This clustering should be interpreted as an exploratory hospital-level classification. DNT, door-to-needle time; DPT, door-to-puncture time; PRT, puncture-to-recanalization time.

To further explore variations in stroke care performance, we conducted a cluster analysis based on six key indicators: DNT compliance rate, DPT compliance rate, PRT compliance rate, intravenous thrombolysis rate within 4.5 h (IVT_4.5h), endovascular therapy rate within 6 h (EVT_6h), and in-hospital mortality after EVT. Using these variables, the 33 hospitals were grouped into three clusters using K-means clustering for each year from 2021 to 2024, and the results were visualized via radar plots (Figure 4). The three-cluster solution provided an exploratory classification with reasonable statistical separation and clinical interpretability, but should not be considered a definitive structural classification of hospitals.

Across all 4 years, Cluster 3 (green) generally represented hospitals with higher observed process compliance, greater reperfusion accessibility, and lower EVT-related in-hospital mortality. This cluster primarily included large tertiary A-level hospitals such as Chengdu Third People’s Hospital, reflecting the leading role of regional stroke centers in delivering high-quality care. Cluster 1 (red), in contrast, demonstrated significantly lower performance across multiple indicators. These findings suggest a need for urgent improvement in workflow optimization and technical capacity. Hospitals in this cluster were predominantly secondary-level institutions. Cluster 2 (blue) exhibited intermediate performance, with some variation across years and indicators, suggesting partial adherence to process standards and moderate accessibility to reperfusion therapy.

This exploratory cluster-based classification may help identify potential targets for quality improvement, but the resulting hospital typology should be interpreted cautiously and requires validation in future studies.

## Discussion

Rapid reperfusion through IVT, EVT, or a combination of both has become a cornerstone in the management of AIS. According to AHA/ASA guidelines, IVT should be administered within 4.5 h of symptom onset, while EVT (for large vessel occlusion) should ideally occur within 6 h. Numerous studies have shown that earlier recanalization is associated with better functional outcomes. Consequently, key time-based process indicators such as DNT, DPT, and PRT have been established as critical quality metrics in stroke care. According to AHA/ASA guidelines, a DNT of ≤60 min is recommended for at least 50% of patients receiving IVT (15, 16). A large U. S. nationwide cohort study showed that each 15-min reduction in DNT was associated with increased home-time and reduced one-year mortality in patients undergoing combined IVT + EVT (17). Similarly, the DPT time within 90 min was recommended by recent evidence (18). Moreover, maintaining a PRT of ≤60 min is supported by stroke quality metrics as a key performance indicator, with procedural efficiency directly linked to improved recanalization rates and reduced disability (5, 19). Registry data show every 30-min delay from puncture to reperfusion can reduce the odds of 90-day functional independence by approximately 13% (20). Together, these data support the clinical relevance of DNT, DPT, and PRT as time-sensitive performance indicators in acute stroke care.

In this multicenter retrospective study of 33 hospitals in Chengdu from 2021 to 2024, we identified important hospital-level patterns and disparities in process quality, treatment accessibility, and EVT-related in-hospital outcomes for AIS. While DNT compliance remained relatively stable and high across all subgroups, more variation was observed in DPT and PRT compliance, particularly among hospitals with lower administrative levels or without stroke center certification. Stroke centers were associated with higher observed adherence to several process indicators, especially DNT and PRT. Although IVT accessibility within 4.5 h was generally equitable across hospital levels and regions, EVT within 6 h showed persistent disparities. Tertiary A-level hospitals, urban hospitals, and certified stroke centers were associated with higher observed EVT rates across all years. These differences may reflect variations in interventional capability, referral pathways, patient triage, or case complexity, which were not fully captured in the current dataset.

Regarding EVT-related in-hospital mortality, tertiary A-level hospitals and stroke centers showed lower and more stable observed rates, despite their likely higher case severity. In contrast, non-tertiary and non-stroke center hospitals exhibited greater year-to-year fluctuations, including a marked mortality spike in 2023, which may be related to unmeasured factors such as differences in case severity, patient selection, or referral patterns. However, these explanations remain speculative, as detailed individual-level clinical data (e.g., stroke severity) were not available in the current study. Importantly, in-hospital mortality following EVT represents only a limited aspect of clinical outcomes and should be interpreted as a subgroup-specific in-hospital outcome rather than a comprehensive outcome measure. In contemporary stroke research, functional outcomes—such as the mRS at 90 days—are considered the gold standard for evaluating treatment effectiveness. However, such data, along with other safety indicators including sICH and IVT-related complications, were not available in the current dataset. Therefore, the outcome-related findings of this study should be interpreted with caution, primarily reflecting differences in severe adverse events rather than overall patient-centered outcomes. Finally, exploratory cluster analysis provided a descriptive view of hospital-level performance patterns based on six key indicators. Hospitals in Cluster 3—predominantly tertiary A-level hospitals and stroke centers—showed higher observed process compliance, greater treatment accessibility, and lower EVT-related in-hospital mortality, whereas those in Cluster 1—mainly non-stroke centers and secondary-level hospitals—showed lower observed performance across several indicators. These findings are consistent with recent national studies examining hospital-level disparities in stroke management. For instance, Chen et al. reported substantial geographic variations in in-hospital mortality and EVT use among IS patients across 1,267 tertiary hospitals in China, with significantly lower EVT utilization in Western and Northeastern regions compared to Eastern regions (21). Evidence from other time-sensitive vascular emergencies also suggests that hospital level may influence access to reperfusion services; for example, Zhang et al. (22) reported lower reperfusion rates in secondary hospitals than in tertiary hospitals among patients with ST-elevation myocardial infarction in Central China, although in-hospital mortality did not differ significantly. Unlike prior national registries focused mainly on tertiary hospitals, our study captured a broader hospital spectrum—including secondary hospitals and non-stroke centers—thus revealing grassroots-level stroke care capacity and institutional disparities within a single metropolitan area.

These observations prompt a deeper examination of the underlying factors contributing to the observed disparities. The relatively uniform performance in DNT may reflect the widespread adoption of standardized in-hospital stroke protocols and heightened awareness across hospital levels and center types. In contrast, the greater variability in PRT compliance may reflect differences in procedural complexity, availability of experienced neurointerventionists, and access to advanced imaging or hybrid operating suites—resources more commonly found in tertiary hospitals and certified stroke centers. The persistent gap in EVT accessibility between tertiary A-level hospitals and their non-tertiary counterparts, as well as between stroke centers and non-stroke centers, highlights systemic limitations in referral efficiency, interhospital coordination, and catheterization lab coverage. Although overall in-hospital mortality remained low, the marked increase observed in non-stroke centers and non-tertiary hospitals in 2023 should be interpreted cautiously. Without patient-level risk adjustment, this fluctuation may reflect case-mix shift, referral of higher-risk patients, differences in baseline stroke severity, or instability caused by a small number of EVT cases, rather than differences in hospital performance alone. Compared to international registries, our findings align with those observed in other low- and middle-income countries (LMICs), where significant disparities in resource allocation, specialist availability, and acute stroke service delivery persist. For example, a systematic review highlighted stark differences in access to stroke units, trained personnel, and reperfusion therapies between LMICs and high-income countries, contributing to higher mortality and disability rates in the former (23). Similarly, global burden of disease analyses have shown that LMICs bear a disproportionate share of stroke incidence and care inequalities, underscoring the ongoing challenge of achieving equitable stroke systems of care worldwide (24).

Given the identified disparities in process compliance and treatment accessibility, targeted strategies are essential to enhance acute stroke care. First, lower-tier hospitals—especially those in Cluster 1—should receive infrastructure investment and workforce training, including stroke team development, acquisition of neurointerventional equipment (e.g., biplane DSA, perfusion software), and simulation-based EVT training to address low compliance with PRT and DPT. Second, regional stroke alliances should be strengthened through a hub-and-spoke model, enabling tertiary centers to support peripheral hospitals via standardized referral, telemedicine, and shared resources. Monitoring interhospital transfer times should be part of this strategy. Third, key performance indicators (DNT, DPT, and PRT) should be integrated into real-time quality dashboards to guide benchmarking and continuous improvement through regular audits and feedback. Finally, these efforts should align with national initiatives—such as the Stroke Prevention and Control Project and stroke center certification—and be supported by performance-based incentives to promote equitable, high-quality reperfusion care (25).

This research delivers important findings on the current status and regional disparities of reperfusion quality, but multiple limitations are acknowledged. First, it was conducted exclusively in Chengdu, Sichuan province, which may limit the generalizability of our findings to other regions with different healthcare infrastructures. Second, as a retrospective analysis, the study is subject to potential selection bias and incomplete data reporting. Third, the current dataset lacked individual-level clinical and pathway-related variables required for risk adjustment, including baseline NIHSS score, imaging characteristics, occlusion site, onset-to-door time, transfer status, triage pathway, anesthesia strategy, and other indicators of case complexity. Therefore, differences in EVT-related mortality or process performance across hospital categories should be interpreted cautiously, as they may reflect variations in patient selection, referral patterns, or clinical acuity rather than hospital performance alone. Fourth, key patient-centered outcomes were unavailable, including post-hospital discharge disposition, 90-day mRS, stroke recurrence, readmission rates, long-term mortality, symptomatic intracranial hemorrhage, and other reperfusion-related complications. Consequently, in-hospital mortality following EVT was analyzed only as an in-hospital outcome measure among EVT-treated patients. This measure cannot fully capture functional recovery, quality of survival, or the overall effectiveness and safety of reperfusion therapy across the entire AIS cohort. Future prospective, multi-provincial studies incorporating both patient- and system-level data, detailed clinical severity and imaging variables, treatment pathways, and longitudinal functional outcomes are needed to validate and expand upon these findings.

## Conclusion

This multicenter study reveals persistent disparities in AIS reperfusion care across 33 hospitals in Chengdu from 2021 to 2024. Stroke center status was associated with higher observed process compliance, accessibility, and EVT-related in-hospital outcome indicators; however, residual confounding cannot be excluded because patient-level risk adjustment was unavailable. Despite overall improvements, gaps remained by hospital level and region, particularly in PRT compliance and EVT coverage. Cluster analysis highlighted institutional heterogeneity, underscoring the need for targeted system-level improvements. Strengthening infrastructure, personnel training, and stroke network integration—especially in non-tertiary A-level and non-stroke center hospitals—is essential to advancing equitable and effective stroke care in western China.

## Data Availability

The raw data supporting the conclusions of this article will be made available by the authors, without undue reservation.
